# Surgical management of acquired bladder diverticula in adult men: a scoping review

**DOI:** 10.1007/s00345-026-06633-5

**Published:** 2026-07-31

**Authors:** Hasim Bakbak, Gurpremjit Singh, Theodora Maria Zavos, Aravindh Rathinam, Tomas Panqueva Baena, Ahmad Abdelaziz, Jonathan Elliot Katz, Robert Marcovich, Alan J. Wein, Hemendra Navinchandra Shah

**Affiliations:** 1 Desai Sethi Urology Institute, University of Miami Miller School of Medicine, Miami, FL USA; 2 University of Louisville School of Medicine, KY Louisville, USA; 3 Miller School of Medicine, University of Miami, Miami, FL USA

**Keywords:** Acquired bladder diverticula, Benign prostatic obstruction, Diverticulectomy, Robotic diverticulectomy, Laparoscopic diverticulectomy

## Abstract

**Purpose:**

To provide an overview of surgical management strategies for acquired bladder diverticulum (BD) in men with associated benign prostatic obstruction (BPO).

**Methods:**

We conducted a scoping review of studies between January 1, 1975, and September 1, 2025, reporting surgical management of acquired BD in men > 45 years. Studies evaluating BD treatment alone or combined with BPO surgery were included. Evidence was stratified by surgical approach.

**Results:**

Forty-four studies encompassing 458 patients were included of which 344 (75.1%) underwent concomitant BPO surgery, most commonly transurethral resection of the prostate. Transurethral (*n* = 112), laparoscopic (*n* = 93), robotic (*n* = 120), and open (*n* = 133) approaches demonstrated comparable baseline age, whereas the robotic cohort had the largest prostate volume (68.4 cc) and diverticular size. Concomitant BPO treatment was most frequent in the transurethral (88.4%) and open (85%) groups, and least common in robotic cases (48.3%) where it was more often staged. Open series generally reported greater blood loss and longer catheter duration and hospital stay. Major complications (Clavien–Dindo ≥ 3) were uncommon (2.2%), ranging from 0% (transurethral) to 3.2% (laparoscopic), whereas minor complications occurred in 5%. All approaches demonstrated functional improvement.

**Conclusions:**

Although comparative effectiveness data remain limited, all approaches to bladder diverticulectomy demonstrate substantial functional improvement with low complication rates, with the transurethral approach showing low operative time and morbidity descriptively. However, the available evidence is predominantly low level and highly heterogeneous, so these findings should be interpreted as descriptive.

**Supplementary Information:**

The online version contains supplementary material available at 10.1007/s00345-026-06633-5.

## Introduction

Bladder diverticula (BD) are outpouchings of bladder mucosa between detrusor fibers [1]. They may be congenital or acquired, with acquired diverticula comprising ~ 90% of cases [1]. Congenital diverticula include all layers of the bladder wall, whereas acquired diverticula are pseudodiverticula composed of urothelium. [2–3] Acquired BD are most often associated with bladder outlet obstruction (BOO), particularly benign prostatic obstruction (BPO), with ~ 70% reported as secondary to BPO [4]. Less common causes include neurogenic bladder, detrusor-sphincter dyssynergia, and prior bladder surgery [1, 5]. The incidence of acquired BD attributed to BPO is estimated at 6%, and approximately 13% across all causes of BOO. [6–7]

Most BD are asymptomatic, but some present with lower urinary tract symptoms (LUTS), recurrent urinary tract infections (RUTIs), bladder stones (BS), and urinary retention, particularly when diverticula are large or have a narrow neck [8–10]. Diverticular size is a major determinant with diameters > 5.15 cm associated with urinary retention [11]. BD are often detected incidentally on imaging [10–12]. Although guidelines do recommend that all men with BD be evaluated for BOO, no specific guidelines exist. [13–14] While highlighting absence of high-level evidence, Powell et al. noted the shift in management of BD from routine excision toward surveillance [4]. For asymptomatic diverticula, conservative management after treatment of BOO is recommended [13]. Surgical management is typically reserved for patients presenting with RUTI, BS, LUTS and hydronephrosis [5, 13]. Operative approaches include transurethral, robotic, laparoscopic, and open techniques [1]. The choice of surgical approach appears to depend on anatomy, surgeon experience, and resources, and may be performed for BD alone or concurrently with BPO relief [15]. 

High-level evidence guiding management of BD remains limited, with most data derived from case reports (CR), small case series (CS), and retrospective cohorts (RC), often in malignancy-focused or pediatric populations [16–18]. A recent review concluded diverticulectomy remains the reference treatment, but concomitant outlet surgery and small heterogeneous series limit firm conclusions [19]. Building on this, no review has yet synthesized the surgical management of BD specifically in adult men with concomitant BPO in an approach-stratified, pooled manner. Accordingly, this scoping review summarizes contemporary surgical options in an approach-stratified manner, providing a practical framework to support shared decision-making between urologists and patients.

## Methods

### Search strategy and inclusion criteria

A search was conducted on September 1, 2025 using PubMed (MEDLINE) database, covering studies from January 1, 1975 to September 1, 2025. Embase search was also performed but did not identify any additional eligible studies. The search query was (“bladder diverticulum” OR “bladder diverticula” OR “diverticulectomy” OR “bladder diverticulectomy” OR “bladder diverticulum excision” OR “surgical treatment of bladder diverticulum”) The search was restricted to English-language, full-text articles. Duplicate records were removed before screening. Reference lists of included studies and relevant reviews were additionally hand-searched. Two authors (HB, TMZ) screened the records, blinded to each other’s decisions. Disagreements were resolved by consensus; if consensus could not be reached, a third senior author (HNS) adjudicated. Studies were included if they reported on men > 45 with surgical management of BD, either with concomitant BPO procedure or diverticular treatment alone. The > 45-year threshold was pre-specified to capture the population in whom acquired, BPO-related diverticula predominate. Studies involving pediatric patients with congenital diverticula or those focusing on diverticula treated for oncologic purposes were excluded. In studies that included mixed populations, we assessed whether subgroup data were reported separately. We included studies explicitly presenting individual subgroup outcomes. If subgroup-specific data were not provided, we evaluated the proportion of these non-eligible cases within the total study population. Studies in which such cases accounted for less than 10% of the cohort were retained, whereas those exceeding were excluded. This threshold was pre-specified in the protocol as a conservative limit, chosen to minimise contamination of the synthesis while avoiding loss of data. Because the few non-target patients who remained could not always be separated from aggregate data, their influence was assessed in a sensitivity analysis (Supplementary Table 5). Two authors (HB, TMZ), later independently extracted the data into a spreadsheet. Because most studies were CS or CR, risk-of-bias tools were not applicable; instead, studies were appraised with 20-item IHE Quality Appraisal Checklist [20]. (Supplementary Table 6) The protocol was initially registered in PROSPERO as a systematic review (CRD420251088810); following data extraction, the heterogeneous, non-comparative nature of the evidence led us to adapt to a scoping review, which is reported in accordance with the PRISMA-ScR guideline.

### Stratification by operative approach

Studies were categorized by the approach as transurethral, laparoscopic, robotic, or open. Within each subgroup, outcomes were synthesized with respect to efficacy, safety, feasibility, and peri-/postoperative results. When concomitant treatment for BPO was performed, it was recorded and reported according to its corresponding modality. For each study the following variables were extracted: age, baseline PSA, prostate size, indication for surgery, sample size, number with BPO, number with BD, BPO treatment modality, BD treatment modality, symptom scores, peak flow (Qmax), post-void residual (PVR), diverticular size, complication rates, bleeding, catheterization, operative time, length of stay (LOS), and follow-up protocols. Weighted means (range) were calculated when applicable. For this review, diverticular resolution was defined as radiographic disappearance, or where so specified by the original authors. Clinical success was defined as postoperative improvement in LUTS or functional parameters.

## Results

A total of 44 studies encompassing 458 patients with acquired BD were included, comprising 7 transurethral-only, 13 laparoscopic-only, 13 robotic-only, 5 open-only, and 6 combined-approach studies (supplementary Fig. 1). Early literature was dominated by open and transurethral techniques, followed by the emergence of laparoscopy in the 1990s, which became predominant by the mid-2000s. Over the past decade, robotics have shown rapid growth (Supplementary Fig. 2).

Most studies were CS (*n* = 23), with fewer retrospective comparative studies (*n* = 6) and CR (*n* = 15). Among all patients, 403/458 (88%) had BD secondary to BPO, and 344/458 (75.1%) underwent concomitant surgical BPO treatment, most commonly transurethral resection of the prostate (TURP) while the remainder were treated in a staged fashion. Concomitant BPO management was most frequent in the transurethral (88.4%) and open (85%) groups and least common in robotic cases (48.3%) where staged procedures predominated. Indications were primarily LUTS and RUTI. Diagnostics evolved, shifting from cystography toward cross-sectional imaging often supplemented by cystoscopy (Table 1).

Perioperative reporting was heterogeneous. The weighted baseline age was similar. Diverticulum size ranged from 5.9 to 7.9 cm, and prostate volumes were largest in the robotic cohort (68.4 cc). The mean weighted operative time was shortest for transurethral surgery (86 min) and longest for laparoscopic cases (196 min). LOS generally remained under two weeks and shortened over time (Supplementary Fig. 3a), while catheterization typically ranged from 1-2 weeks. Estimated blood loss was inconsistently reported but tended to be lower in minimally invasive series and higher in open cohorts. Open surgery demonstrated the greatest blood loss (3.9 g/dL hemoglobin drop or 425 mL) and longest catheter duration (14.9 days) and LOS (10.8 days). Major complications (Clavien–Dindo ≥ 3) were uncommon overall (2.2%), ranging from 0% (transurethral) to 3.2% (laparoscopic), while minor complications occurred in 5%, with a modest increase in reported events over time, likely reflecting improved reporting practices (Supplementary Fig. 3b). Functional outcomes demonstrated consistent improvement across approaches, including increases in Qmax (+ 8.4 to + 15.2 mL/s), > 85–90% reductions in PVR, and marked improvements in symptom scores (Supplementary Fig. 3c, Table 1). Diverticular resolution was reported in 70–80% of transurethral cases, and in most minimally invasive cases.

A sensitivity analysis excluding the 7 non-eligible patients left all pooled estimates essentially unchanged (Supplementary Table 5). Methodological appraisal of all 44 studies showed variable reporting: newer studies described selection, procedures, outcomes, follow-up, and complications more clearly than older ones.

**Table 1:** Pooled Summary of Baseline Study and Patient Characteristics, Perioperative Outcomes Stratified by Treatment Approach

Overall Evidence	
Number of Patients	458
Number of Acquired BD Secondary to BPO	403 (88%)
Number Underwent Concomitant BPO Surgery	344 (75.1%)
Number of Major Complications (CD ≥ 3)	10 (2.2%)
Number of Minor Complications (CD ≤ 2)	23 (5%)

Patient Characteristics, Perioperative Outcomes Stratified by Treatment Approach				
	Transurethral group	Laparoscopic group	Robotic group	Open group
*Study characteristics*				
Number of studies (n)#	9	17	15	9
Era of publication	1977–2025	1992–2020	2007–2024	1977–2021
Total patients with BD (n)	112	93	120	133
Total patients with BPO (n)	103 (91.9%)	86 (92.4%)	98 (81.6%)	116 (87.2%)
Study design	2RC, 6CS, 1CR	3RC, 7CS, 7CR	2RC, 9CS, 4CR	4RC, 2CS, 3CR
*Baseline data*				
Age (Years)	68.4 (65–75)	68.6 (65–75)	65.7 (60–75)	65 (60–75)
BD diameter (cm)	7.4 (7–8.5)	5.9 (4.5–7)	7.9 (7–12.5)	6.3 (4.5–7)
Prostate size (cc)	52.9	60.2 (30–70)	68.4 (55–75)	52.5 (35–70)
Primary indication	LUTS/RUTI	LUTS/RUTI/SBD	LUTS/RUTI	LUTS/Retention
*Surgical strategy*				
Underwent BPO (% of cohort)	91.9%	92.4%	81.6%	87.2%
Concomitant BPO (% of cohort)	88.4%	79.6%	48.3%	85%
Predominant BPO approach	TURP	TURP, TUEP	RSP, RARP, TURP	OSP, Enucleation
*Perioperative outcomes*				
Operative time (combined, min)	86 (60–300)	196 (120–240)	165 (120–240)	148 (120–180)
Est. blood loss (Hb)	0.8–1.76 g/dL drop	< 50-150 ml	50–150 ml	3.9 g/dl drop/425 ml
Catheter duration (days)	9.8 (3–15)	9.1 (5–15)	7.1 (1–25)	14.9 (3–44)
Length of stay (days)	5.6 (0.8-9)	6.2 (2–10)	3.6 (1–7)	10.8 (3.2–19.6)
*Complications*				
Major (Clavien Dindo ≥ 3)	0	3.2% (*n* = 3)	2.5% (*n* = 3)	3% (*n* = 4)
Minor (Clavien Dindo ≤ 2)	3.6% (*n* = 4)	1.1% (*n* = 1)	9.2% (*n* = 11)	5.3% (*n* = 7)
*Functional outcomes*				
Qmax(ml/s) (Pre to post)	+ 8.4 (3.1–11.5)	+ 11.3 (5–20)	+ 12.2 (8.5–25.2)	+ 15.2 (6.1–24.8)
PVR reduction (ml) (Pre to post)	> 90% (373 to 8–32)	> 90% (> 150 to negligible)	> 85% (164–741 to 30–60)	> 85% (425 to 49)
IPSS reduction (Pre to post)	55% (50–65% decrease)	60% decrease	15 points (19–25 to 5–8)	13 points (19 to 6)
*Clinical Success*				
Diverticula resolution	70–80%	Mostly resolved	NA	NA
Radiographic follow-up	Cystogram/US	CT/ US/ Cysto	CT/ US/ VCUG	Cystogram/ MRI

### Open approach (Supplementary Tables 1a-1b)

Nine studies (4 RC, 2 CS, 3 CR) included 133 patients undergoing open diverticulectomy. Most (87.2%) received surgical treatment of BPO of which 97.4% (85% of cohort) concomitantly, commonly open simple prostatectomy or TURP. Age mainly clustered around 60–75 and diverticular size clustered around 4.5–7 cm. Mean weighted operative time was 148 min (120–180), with greater blood loss compared to minimally invasive approaches (mean hemoglobin drop 3.9 g/dL or approximately 425 mL). Catheterization averaged 14.9 days, and LOS was also prolonged at 10.8 days. Major complications occurred in 3% (*n* = 4), and minor complications in 5.3% (*n* = 7). Functional outcomes were less consistently reported, reflecting earlier eras, though available data demonstrated meaningful improvements in flow and symptom burden. Interpretation is limited by heterogeneity.

### Transurethral approach (Supplementary Tables 2a-2b)

Nine transurethral studies (2 RC, 6 CS, 1 CR) and two comparative studies with open surgery were included, comprising 112 patients. Most (91.9%) underwent surgical BPO treatment, 96.1% (88.4% of cohort) concomitantly, predominantly TURP. Patients were typically aged 65–75 years, with diverticula measuring in 7–8.5 cm range. Indications were primarily LUTS and RUTI. The weighted mean operative time was 86 min (60–300), with modest blood loss (0.8–1.76 g/dL hemoglobin drop). Catheterization averaged 9.8 days and LOS 5.6 days. Major complications did not occur (0%), and minor complications occurred in 3.6%. Functional outcomes demonstrated meaningful improvement, including a mean Qmax increase of 8.4 mL/s, > 90% reduction in PVR, and 50–65% reduction in symptom scores. Approximately 70–80% of patients achieved diverticular resolution. Interpretation of outcomes remains influenced by concomitant BPO procedures and practice variability.

### Laparoscopic approach (Supplementary Tables 3a-3b)

Seventeen studies (3 RC, 7 CS, 7 CR) comprising 93 patients were included. Most (92.4%) underwent surgical BPO treatment, typically transurethrally rather than laparoscopic prostatectomy with 86% (79.6% of cohort) concomitantly. Patients were commonly aged 65–75 years, with diverticular sizes ranging from 4.5 to 7 cm. The weighted mean operative time was 196 min (120–240) and estimated blood loss was low (< 50–150 mL). Catheter duration averaged 9.1 days (5–10), and LOS was approximately 6.2 days. Major complications occurred in 3.2% (*n* = 3), while minor complications were reported in 1.1% (*n* = 1). Functional outcomes were favorable, with Qmax increasing by 11.3 mL/s, > 90% reduction in PVR, and approximately 60% reduction in symptom score. Most studies reported radiographic or clinical diverticular resolution on follow-up imaging.

### Robotic approach (Supplementary Tables 4a-4b)

Fifteen studies (2 RC, 9 CS, 4 CR) reported outcomes for 120 patients undergoing robotic diverticulectomy. Patients were generally 60–75 years old and robotic cohorts included larger diverticula (mean 7.9 cm, range 7–12.5 cm), with weighted mean prostate volumes averaging 68.4 cc. Surgical BPO treatment was performed in 81.6% amongst which only 59.1% (48.3% of cohort) concomitantly. Choice of BPO surgery was more heterogeneous comprising both robotic prostatectomy and transurethral approaches. Mean weighted operative time was 165 min (120–240), with estimated blood loss ranging from 50 to 150 mL. Catheterization averaged 7.1 days (5–15), and LOS was approximately 3.6 days (3–7). Major complications occurred in 2.5% (*n* = 3), while minor complications were reported in 9.2% (*n* = 11). Functional outcomes demonstrated meaningful improvement, with Qmax increasing by 12.2 mL/s, > 85% reduction in PVR, and substantial symptom score improvement. Reported diverticular resolution was high, with acceptable overall safety.

## Discussion

### Interpretation of overall evidence

Literature on the surgical management of BD remains limited and methodologically heterogeneous, and reporting is inconsistent. The diagnostic pathway relied on cystography earlier, with a shift toward CT and US, followed by cystoscopy; this evolution may have affected case ascertainment and reported diverticular characteristics. Clinically, the data reinforce that acquired diverticula are commonly a consequence of BPO, and a large proportion of patients underwent concomitant BPO surgery, most often TURP, suggesting that diverticulectomy is frequently performed alongside outlet correction rather than as an isolated procedure.

Perioperative outcomes appear acceptable, with LOS generally remaining under two weeks and shortening over time, catheter duration commonly around one to two weeks, and blood loss tending to be lower in minimally invasive series. These improvements likely reflect advances in healthcare delivery and modern technologies. Additionally, earlier eras may have favored longer hospital stays, whereas contemporary practice favors earlier discharge. Major complications were uncommon (2.2%) and minor complications were infrequent (5%), and the modest rise in cumulative complications likely reflects improved capture and reporting rather than a true increase. Functional outcomes generally improved across approaches, with substantial reductions in PVR and improvements in symptom scores; however, because outlet surgery was frequently performed and outcomes were variably reported, the relative contribution of diverticulectomy versus BPO relief cannot be reliably disentangled from the existing evidence as also noted in the recent EAU review [19].

### Open approach

Early CS were published in the 1940s by Thompson and Kimbrough, reflecting the historical role of open surgery as the standard management. [21–22] Later, Firstater et al. used a transvesical approach to perform submucosal dissection to avoid rectal injuries and damage to ureters and vas deferens [23]. In contemporary urology, however, the open approach has largely declined with the widespread adoption of minimally invasive techniques.

The principal advantage of the open approach is universal availability, as it does not require advanced equipment. However, longer hospitalization, extended catheterization, and prolonged recovery have limited its role in contemporary practice.

### Transurethral approach

Transurethral era of BD management began in 1977 with Orandi’s description of mucosal fulguration [24]. Orandi’s concept was to treat the diverticulum by fulgurating the diverticular mucosa, with the goal of shrinking the sac and improving emptying [24]. Posta described incision of diverticular neck to improve drainage and reduce stasis [25]. Subsequently, Clayman et al. combined these two approaches [26]. Pacella took this technique further in 2019, using the same concept by a rollerball and bipolar energy [27]. Most recently, Rathinam et al. combined resection of diverticular neck and fulguration of entire diverticular mucosa using bipolar resectoscope with HoLEP [10]. 

Transurethral management offers feasibility and integrates naturally with BPO surgery, as diverticulum-directed maneuvers are typically brief and performed concomitantly [15]. Conceptually, the major limitation of this strategy is mechanistic. BD represents a structural defect of the bladder wall, and transurethral techniques typically do not remove the diverticulum; rather, they aim to reduce urinary stasis by modifying the diverticular neck and treating mucosa [28]. However, there are reports of complete radiographic resolution of BD with this approach that led to renewed interest in this approach [9, 27]. Among the reviewed approaches, it showed the shortest operative time and no major complications. Importantly, these are descriptive observations rather than a comparative claim.

### Laparoscopic approach

Laparoscopy begins with Parra et al. in 1992, who adopted transperitoneal approach and used simultaneous cystoscopy to locate the diverticular neck [29]. It represented the first widely adopted minimally invasive approach that enabled true excision of the diverticular sac and closure of the defect [29]. Later on, Jarret et al. utilized a similar approach but localized the diverticulum by combination of imaging and catheterization [30]. Subsequently, Porpiglia inflated the balloon of the catheter inside to distend the diverticulum [31]. In 2006, Shah et al. initiated an extraperitoneal approach [32]. 

This approach enables definitive excision with favorable perioperative outcomes and represents a cost-effective alternative to robotics where robotic systems are unavailable. [33–34] However, reduced dexterity in the deep pelvis often necessitates separate transurethral management of BPO rather than a combined laparoscopic outlet procedure. [35–36]

### Robotic approach

Robotic diverticulectomy was first reported by Berger et al. in 2006 [37]. The robotic approach shares the same objective as laparoscopy with excision of the sac and closure of the defect. The earliest series by Myer et al. describes a transperitoneal approach and utilized a catheter to distend the diverticulum [38]. In the late 2010s, technique evolved towards extravesical approach [39]. Following adjustments mainly focused on different catheter applications and varying suturing techniques [40–42]. 

Robotics allows definitive excision with enhanced dexterity and visualization, facilitating management of larger or more complex diverticula. [35–36] Several series report combined diverticulectomy with robotic simple prostatectomy, although practices vary. The lower concomitant rate in the robotic cohort reflects frequent staging of outlet surgery rather than reduced overall treatment. Therefore, concomitant-BPO proportions should not be compared directly, as differences are confounded by case selection and staging practices. Primary limitation remains cost and restricted access to robotic platforms. [33–34]

### Conservative management/Observation for BD

Observation remains appropriate for small, asymptomatic diverticula. Medical optimization of BPO with alpha-blockers, PDE5 inhibitors, and 5-alpha-reductase inhibitors is commonly employed, with escalation reserved for refractory symptoms [11]. A conservative “obstruction-first” strategy is supported by contemporary series demonstrating successful observation following outlet relief alone [43–45]. In practice, diverticulectomy is reserved for persistent symptoms or diverticulum-related complications. The individual studies contributing to the open, transurethral, laparoscopic, and robotic approach-stratified syntheses are detailed in Supplementary Tables 1a–4b [46–66]." 

### Limitations

This review synthesizes heterogeneous, largely non-comparative evidence, including CR and CS, reflecting the rarity of symptomatic BD. Some included cohorts contained a small minority of patients outside the strict target population. However, a sensitivity analysis excluding non-eligible patients yielded essentially identical pooled estimates (Supplementary Table 5), indicating that their inclusion did not meaningfully bias the synthesis. Notably, as BPO surgery was performed concomitantly or in a staged fashion in the large majority of patients, the functional improvements attributed to diverticulectomy are confounded by concurrent BPO relief. Consequently, differences may reflect the heterogeneity of the case selection, and no indirect comparisons between approaches should be drawn. Most available evidence came from small CS and CR, with variable reporting quality. Therefore, the findings should be interpreted descriptively. While conclusions are hypothesis-generating, consistent patterns across the literature provide a useful framework for contemporary clinical decision-making.

## Conclusion

All surgical approaches appear safe and effective in selected patients, although superiority cannot be established from this limited evidence. In practice, selection should be individualized according to diverticular size, symptom burden, associated complications, need for concomitant BPO surgery, surgeon expertise, and local resources. Transurethral management appears to be a pragmatic option in patients undergoing surgery for BPO, offering short operative time, minimal blood loss, no reported major complications, and meaningful functional improvements. Laparoscopic and robotic approaches also provide favorable functional outcomes with acceptable complication rates. Although robotic cohorts often included larger diverticula, major complication rates were low across approaches (0–3.2%), with consistent symptomatic improvement.

However, the evidence is largely derived from RC and CS, introducing potential bias and outcomes should be interpreted as descriptive rather than comparative. Prospective studies are needed to optimize patient selection and effectiveness across surgical strategies. This review does not support a simple formula or strict “standards of care” for surgical management of acquired BD; however, the cumulative findings can still be used to pragmatically guide patient selection, counseling, and shared decision-making. (Supplementary Fig. 4)

## Supplementary Information

Below is the link to the electronic supplementary material.

Supplementary Material 1

Supplementary Material 2

Supplementary Material 3

Supplementary Material 4

Supplementary Material 5

Supplementary Material 6

Supplementary Material 7

Supplementary Material 8

Supplementary Material 9

Supplementary Material 10

## Data Availability

All data analyzed in this review are derived from published studies. The extracted data tables used for synthesis are included in the manuscript and/or supplementary material; additional extraction worksheets are available from the corresponding author upon reasonable request.

## Abbreviations

BD: Bladder diverticulum / bladder diverticula
BPH: Benign prostatic hyperplasia
BPO: Benign prostatic obstruction
BOO: Bladder outlet obstruction
CR: Case report
CS: Case series
RC: Retrospective cohort
CT: Computed tomography
HoLEP: Holmium laser enucleation of the prostate
IPSS: International prostate symptom score
PVR: Postvoid residual volume
LD: Laparoscopic diverticulectomy
LUTS: Lower urinary tract symptoms
OD: Open diverticulectomy
PDE5i: Phosphodiesterase-5 inhibitor(s)
RC: Retrospective comparative study
RD: Robotic diverticulectomy
RSP: Robotic simple prostatectomy
RUTI: Recurrent urinary tract infection(s)
TURP: Transurethral resection of the prostate
US: Ultrasonography / ultrasound
UTI: Urinary tract infection
LOS: Length of stay
