# Supplementary material for: Surgical management of acquired bladder diverticula in adult men: a scoping review
Source: World J Urol. 2026 Jul 31;44(1):537. doi: 10.1007/s00345-026-06633-5 (PMC13427780; doi:10.1007/s00345-026-06633-5)
Supplement: Supplementary file 1 — Supplementary Material 1 [file 345_2026_6633_MOESM10_ESM.docx]

**Supplementary Figure 3a: Temporal trends in length of stay for bladder diverticulectomy (1977 - 2025)**

**
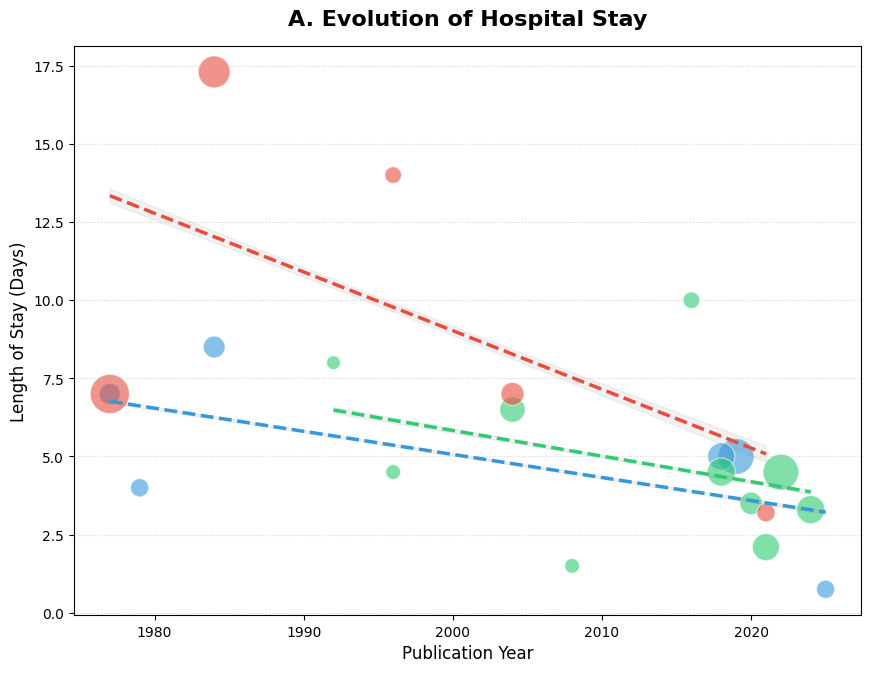
**

**Supplementary Figure 3b: Temporal trend in evolution of complications for bladder diverticulectomy (1977-2025)**

**
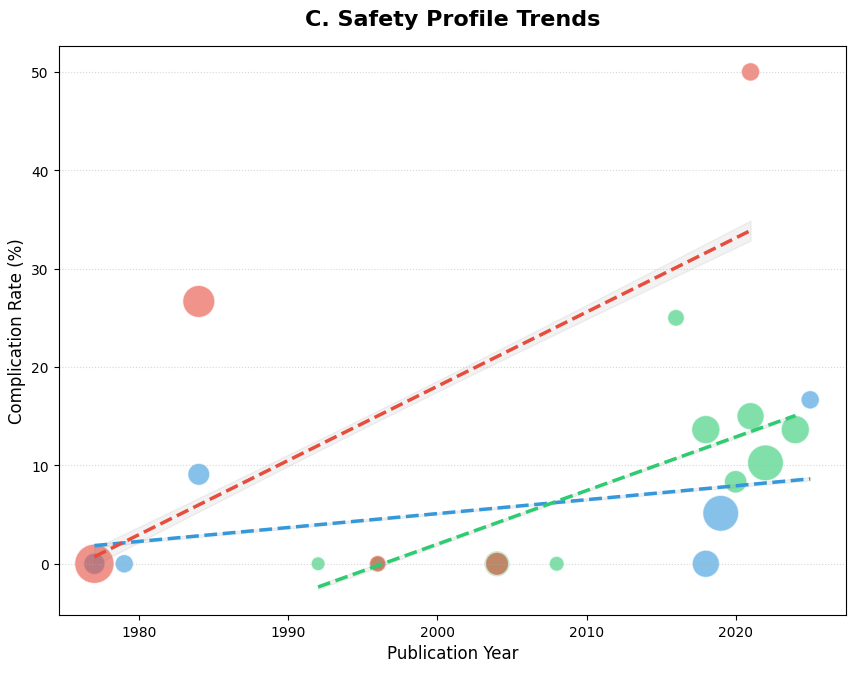
**

**Supplementary Figure 3c: Temporal trend for improvement in Qmax for bladder diverticulectomy**

**(1977-2025)**

**
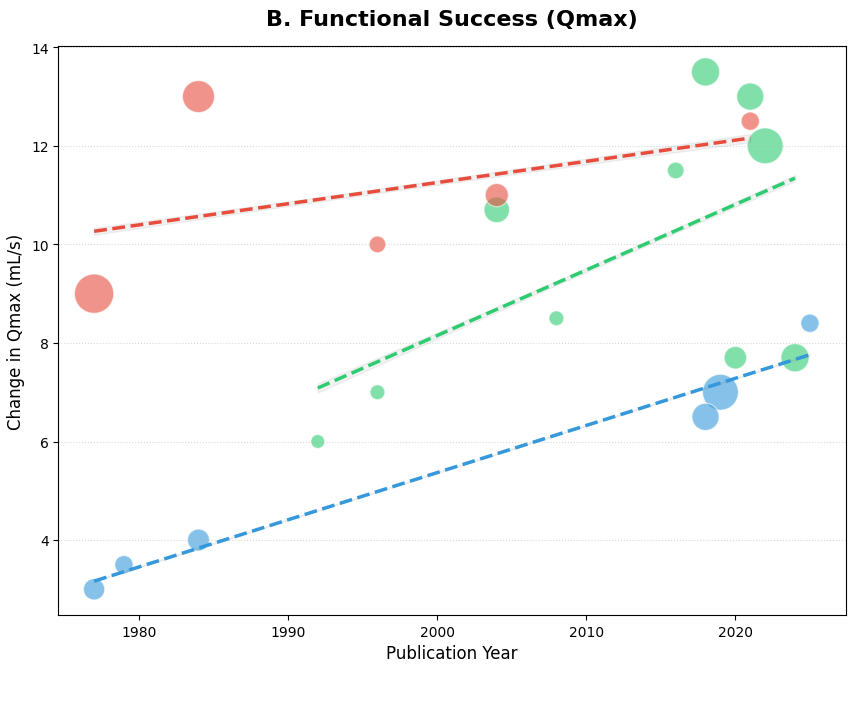
**
