# Supplementary material for: Surgical management of acquired bladder diverticula in adult men: a scoping review
Source: World J Urol. 2026 Jul 31;44(1):537. doi: 10.1007/s00345-026-06633-5 (PMC13427780; doi:10.1007/s00345-026-06633-5)
Supplement: Supplementary file 2 — Supplementary Material 2 [file 345_2026_6633_MOESM1_ESM.docx]

**Supplementary Table 1a. Baseline Characteristics and Demographics of Open Group**

| **Author (Year)** | **Type of Study** | **Sample Size (BD)** | **Sample Size (BPO)** | | **Treatment Approach (BPO)** | **Diagnostics** | **Age** | **Baseline PSA (ng/dl)** | **Prostate Size (cc)** | **Indication of Surgery** | **BD Size** | | **IPSS** | **Qmax** | **PVR** |
| --- | --- | --- | --- | --- | --- | --- | --- | --- | --- | --- | --- | --- | --- | --- | --- |
| Liu et al. (2021)  [OD Subgroup] [46] | RC | 6 | 4^$^ | | TURP, OSP | Imaging + Cystoscopy | 73  (48 - 82)# | NA | NA | RUTI, SBD, LUTS | 7.8  (4-11)* | | 19* | NA | 425 (248)* |
| Iscaife et al. (2018) [ALL Patients Including LD] [11] | RC | 29 | 29^$^ | | NA | CT | 67.2 (12.7) AUR (+) 68.5 (10.4) AUR (-)* | 9.6 (21) AUR (+) 3.5 (5.5) AUR (-)# | 70.7 (58.2) AUR(+), 50.1 (30.2) AUR(-)* | LUTS | 6.8 (2.5) AUR (+)  4.5 (2) AUR(-)* | | NA | 7.9 (7.1) AUR (+) 6.1 (4.9) AUR (-)* | NA |
| Michelotti et al. (2008) [47] | CR | 1 | 1^^^ | | TURP, OSP | CT + Cystogram | 61 | NA | NA | HUN | NA | | NA | NA | NA |
| Porpiglia et al. (2004)  [OD Subgroup] [31] | RC | 13 | 13^$^ | | OSP | US / CT /  RUG / VCUG | NA | NA | 35  (20 - 45)* | RUTI, LUTS | NA | | NA | NA | NA |
| Iselin et al. (1996)  [OD Subgroup] [48] | CS | 4 | 4^$^ | | TURP | Cystoscopy / Cystography | NA | NA | NA | NA | 4.6  (2 - 7)* | | NA | NA | NA |
| Gong et al. (1996) [49] | CR | 1 | 1 | | Medical | Cystogram +  MRI | 75 | NA | NA | Mass Effect | 10 | | NA | NA | NA |
| Jarow et al. (1988) [50] | CR | 1 | 1^^^ | | TURP | Cystogram | 63 | NA | NA | Retention | 11 | | NA | NA | NA |
| Clayman et al (1984)  [OD Subgroup] [26] | RC | 30 | 16^$^ | | OSP | Cystogram | 63 (OD) 68 (OD+OSP) | NA | NA | RUTI, LUTS | NA | | NA | NA | 300 (OD) 400(OD+  OSP) * |
| Firstater et al. (1977) [23] | CS | 48 | 47^$^ | | Enucleation | Cystogram + Pyelogram | 62  (8 - 87)* | NA | NA | LUTS, Poor Emptying | NA | | NA | NA | NA |
| Overall [N=9]  (1977 – 1996) | 4 RC  2 CS  3 CR | 133 | 116  113^$^ | | NA | 5 Cystogram  3 CT  2 Cystoscopy | Clustering  60-75 | NA | NA | 5 LUTS  3 RUTI | Clustering  4.5-7 | | NA | NA | NA |
| This *=Mean (SD/Range) | | | | #= Median (IQR/Range) | | | | $ = Concomitant | | | | ^ = Prior/ Staged | | | |

(OD: Open Diverticulectomy; LD: Laparoscopic Diverticulectomy; RC: Retrospective comparative; CS: Case series; CR: Case report; BD: Bladder diverticulum; BPO: Benign prostatic obstruction; TURP: Transurethral Resection of the Prostate; OSP: Open simple prostatectomy; CT: Computed Tomography; US: Ultrasonography; RUG: Retrograde Urethrography; VCUG: Voiding Cystourethrography; MRI: Magnetic Resonance Imaging; RUTI: Recurrent Urinary Tract Infection; SBD; Bladder Stone; LUTS: Lower Urinary Tract Symptoms; HUN: Hydroureteronephrosis; AUR; Acute Urinary Retention; IPSS: International Prostate Symptom Score; PVR: Postvoid Residual Volume)

**Supplementary Table 1b. Perioperative and Postoperative Outcomes of Open Group**

| **Author (Year)** | **BD Size** | **IPSS** | **Qmax** | **PVR** | **Major Complications (CD≥3)** | **Minor Complications (CD≤2)** | **Blood Loss** | **Operative Time** | **Duration of Catheter (days)** | **Length of Stay (days)** | **Follow Up** |
| --- | --- | --- | --- | --- | --- | --- | --- | --- | --- | --- | --- |
| Liu et al. (2021)  [OD Subgroup] [46] | NA | 6 | NA | 49 (119)* | 3 | 0 | 283  (150 - 750)* | 175  (69 - 288)* | 44  (16-81)* | 3.2  (2 - 6)* | 9 months (6- 51)^#^ |
| Iscaife et al. (2018) [ALL Patients Including LD] [11] | NA | NA | 24.8 (13.8) AUR (+) 19.6 (9) AUR (-)* | NA | NA | NA | NA | NA | NA | NA | NA |
| Michelotti et al. (2008) [47] | NA | NA | NA | NA | 0 | 0 | NA | NA | NA | NA | 3 Months USG |
| Porpiglia et al. (2004)  [OD Subgroup] [31] | NA | NA | 20  (18 - 24)* | NA | NA | NA | 3.9 (3 - 5) Hgb Drop* | 136  (80 - 165)* | 7 | 9.6 | NA |
| Iselin et al. (1996)  [OD Subgroup] [48] | NA | NA | NA | NA | 0 | 0 | 7.5% (4 -12)* Hct Drop | 104  (75 - 135)* | 14 | NA | 1 |
| Gong et al. (1996) [49] | NA | NA | NA | NA | 0 | 0 | NA | NA | 14 | NA | 12 |
| Jarow et al. (1988) [50] | NA | NA | NA | NA | 0 | 0 | NA | NA | NA | NA | 24 |
| Clayman et al (1984)  [OD Subgroup] [26] | Resolution: 8/14 (57.1%) (OD) 8/13 (61.5%) (OD+OSP) | NA | NA | 75.(OD) 20 (OD + OSP) | 1 (OD + OSP) | 5 (OD) 2(OD+OSP) | 425(OD) 1650 (OD + OSP)* | 156 (OD) 150 (OD + OSP) | 3-5 | 19.6 (OD) 15 (OD + OSP)* | 2-19 |
| Firstater et al. (1977) [23] | Uniformly Good | NA | NA | NA | 0 | 0 | NA | NA | 7 | NA | 12 Pyelogram and Cystogram |
| Overall [N=9] | NA | NA | NA | NA | 4/133 (3%) | 7 (5.3%) | NA | Clustering 120-180 | Clustering  7-14 | 10.8 (3.2-19.6) | NA |
| *=Mean (SD/Range) | | | | | | #=Median (IQR/Range) | | | | | |

(OD: Open Diverticulectomy; LD: Laparoscopic Diverticulectomy; OSP: Open simple prostatectomy; IPSS: International Prostate Symptom Score; PVR: Postvoid Residual Volume; CD: Clavien Dindo)
