# Supplementary material for: Surgical management of acquired bladder diverticula in adult men: a scoping review
Source: World J Urol. 2026 Jul 31;44(1):537. doi: 10.1007/s00345-026-06633-5 (PMC13427780; doi:10.1007/s00345-026-06633-5)
Supplement: Supplementary file 3 — Supplementary Material 3 [file 345_2026_6633_MOESM2_ESM.docx]

**Supplementary Table 4a. Baseline Characteristics and Demographics of Robotic Group**

| **Author (Year)** | **Type of Study** | **Sample Size (BD)** | **Sample Size (BPO)** | **Treatment Approach (BPO)** | | **Diagnostic** | **Age (years)** | **Baseline PSA** | **Prostate Size (cc)** | **Indication of Surgery** | **BD Size** | **IPSS** | | **Qmax** | **PVR** |
| --- | --- | --- | --- | --- | --- | --- | --- | --- | --- | --- | --- | --- | --- | --- | --- |
| Gibson et al. (2024) [39] | CS | 18 | 14^^^ | TURP  HoLEP Urolift RSP | | Cystoscopy | 61.72 (12.54)* | NA | NA | RUTI, LUTS | 9.1 (3.61)* | 20.4 (6.78)* | | 14.62  (7.12)* | 741.55 (995)* |
| Orsini et al. (2024) [42] | CS | 4 | 4^$^ | TURP | | Cystoscopy | 73.5  (70.8 - 76)^#^ | NA | 93  (85-103)^#^ | RUTI, LUTS | 12.3  (9.5 - 14.9)^#^ | 24 (24-25)^#^ | | 8.5  (7.75 - 9.5)^#^ | 165  (150 - 187)^#^ |
| Giannarini et al. (2022) [41] | CS | 16 | 16^$^ | TURP | | US + CT + Cystoscopy | 68 (54-74)^#^ | NA | 64  (52-76)^#^ | NA | 6.9 (5.1- 8,2)^#^ | 25 (21-30)^#^ | | NA | 195  (140 - 210)^#^ |
| Develtere et al. (2022) [61] | CS | 23 | 13^$^ | RSP | | Cystoscopy | 66  (60 - 69)^#^ | NA | NA | RUTI, LUTS | 7 (5.4 - 9.7)^#^ | NA | | NA | PreOp-PostOp   120 (33 - 402)^#^ |
| Liu et al. (2021) [RD Subgroup] [46] | RC | 20 | 9^$^  6^^^ | TURP  Urolift  RSP  RARP | | Imaging + Cystoscopy | 66  (56.5-72.5)^#^ | NA | NA | RUTI, LUTS, SBD | 7.6 (3 - 14)* | 19* | | NA | 425 (248)* |
| Agarwal et al. (2018) [RD+OD] [44] | RC | 2 | 2^^^ | HoLEP | | Imaging + Cystoscopy | 54.6* | 1.2* | 37.6* | Hematuria, Urinary Retention | 5.5 (2.6)* [Includes non-operated BD] | 19.7 (9.5)* [Includes non-operated BD] | | 13* | 433* |
| Ashton et al. (2018) [62] | CS | 3 | 2^^^ | TURP | | CT Urogram | 72  (60 - 83)* | NA | NA | RUTI, LUTS | 10, 11.5, 12 | NA | | NA | 1/3 = 812  2/3 = NA |
| Cacciamani et al. (2018) [63] | CS | 6 | 2^$^  3^^^ | TURP | | Cystogram + US | 66.5  (56.5 -72.5)^#^ | NA | NA | LUTS | 7.1  (5.5 - 9.5)# | NA | | NA | 300  (90 - 395)^#^ |
| Tufek et al. (2016) [40] | CS | 9 | 9^$^ | TURP  BNI | | Cystoscopy + US | 62 (9.8)* | NA | 70 (26)* | LUTS, SBD, Retention | 7.2 (1.6)* | NA | | NA | NA |
| Abreu et al. (2014) [64] | CS | 10 | 3^$^  5^ | TURP  RSP RARP | | US + CT + Cystoscopy | 65  (43 - 73)^#^ | NA | 56  (30-145)^#^ | RUTI, Retention | 7.2  (4.6 - 9.8)^#^ | Median 22  (3 - 31)^#^ | | NA | 269  (110 - 815)^#^ |
| Moreno Sierra et al. (2010) [65] | CR | 1 | 1^^^ | TURP Greenlight | | VCUG | 64 | NA | 60 | RUTI, VUR, LUTS | 7 | 35 | | 10 | NA |
| Kural et al. (2009) [2] | CR | 1 | 1^^^ | PVP | | US + CT | 62 | 3.6 | 69 | LUTS | 8 | NA | | 4 | 600 |
| Magera et al. (2008) [66] | CR | 1 | 1^$^ | RSP | | CT | 66 | 21 | 230 | RUTI, LUTS | 6.3 | NA | | NA | 450 |
| Mmeje et al. (2008) [36] | CR | 1 | 1^^^ | TURP | | CT + Cystoscopy | 75 | 1.4 | 60 | RUTI, LUTS | 14 | NA | | 4 | 400 |
| Myer et al. (2007) [38] | CS | 5 | 2^^^  2^#^ | TURP | | Cystoscopy + US | 71 (61-84)^#^ | NA | NA | RUTI, LUTS | 10.9  (4.7 - 15.8)^#^ | NA | | NA | 346  (71 - 558)^#^ |
| Overall [N=15]  (2007 – 2024) | 2 RC  9 CS  4 CR | 120 | 98  58^$^ | NA | | 10 Cystoscopy  6 CT  5 US | Clustering  60-75 | NA | Clustering  55-75 | 12 LUTS  10 RUTI | Clustering  7-12.5 | Clustering  19-25 | | Heterogeneous | Mostly >100 |
| * = Mean (SD/Range) | | | | | # = Median (IQR/Range) | | | | $ = Concomitant | | | | ^ = Prior/Staged | | |

(RD: Laparoscopic Diverticulectomy; OD: Open Diverticulectomy; RC: Retrospective comparative; CS: Case series; CR: Case report; BPO: Benign prostatic obstruction; RSP: Robotic Simple Prostatectomy; TURP: Transurethral Resection of the Prostate; HoLEP: Holmium Laser Enucleation of the Prostate; CT: Computed Tomography; US: Ultrasonography; RUTI: Recurrent Urinary Tract Infection; SBD; Bladder Stone; LUTS: Lower Urinary Tract Symptoms; IPSS: International Prostate Symptom Score; PVR: Postvoid Residual Volume)

**Supplementary Table 4b . Perioperative and Postoperative Outcomes of Robotic Group**

| **Author (Year)** | **IPSS** | **Qmax** | **PVR** | **Major Complications (CD≥3)** | **Minor Complications (CD≤2)** | **Blood Loss** | **Operative Time** | **Duration of Catheter (days)** | **Length of Stay (days)** | **Follow Up** |
| --- | --- | --- | --- | --- | --- | --- | --- | --- | --- | --- |
| Gibson et al. (2024) [39] | NA | 22.32(10.69)* | 41.4 (28.34)* | 1 | 2 | 75* | 105* | 10.1 (8.27)* | 2.67 (1.94)* | NA |
| Orsini et al. (2024) [42] | 7 (6-8)^#^ | NA | 35 (25 – 42)^#^ | 0 | 0 | 100  (80 – 125)^#^ | 212 (191 – 252)^#^ | 9 (7 – 12)^#^ | 4 (4 – 6)^#^ | 6 months |
| Giannarini et al. (2022) [41] | 5 (5-6)^#^ | NA | 30 (28 – 40)^#^ | 0 | 2 | 20 (15-40)^#^ | 126 (92-167)^#^ | 5 (5-7)^#^ | 6 (6-7)^#^ | 6 months IPSS + PVR |
| Develtere et al. (2022) [61] | NA | NA | 42 (0 – 111)^#^ | 0 | 2 | 250  (28 – 438)^#^ | 140 (120 – 180)^#^ | 2 (1-5)^#^ | 3 (2 – 4)^#^ | 9 months Uroflow + USG^#^ |
| Liu et al. (2021) [RD Subgroup] [46] | 6 | NA | 49 (119)* | 1 | 2 | 100  (25 – 200)* | 184 (57 – 386)* | 12.3 (7 – 14)* | 2.1 (1 – 9)* | 9 (6- 51)^#^ |
| Agarwal et al. (2018) [RD+OD] [44] | 5.8 (4.9)* [Includes non-operated BD] | 25.2 (16)* [Includes non-operated BD] | 113.6 (83.4) [Includes non-operated BD] | 0 | 0 | NA | NA | 1 | NA | 12.2* |
| Ashton et al. (2018) [62] | NA | NA | NA | NA | NA | NA | NA | 2/3 = Day 2 1/3 = Week 3 | 3^#^ | Minimum 14 months |
| Cacciamani et al. (2018) [63] | NA | NA | 57.9  (0 – 150)^#^ | 0 | 1 | 25.8  (0 – 50)^#^ | 5.3 (7 – 15)^#^ | 5.3 (7 – 15)^#^ | 7 (4.7 – 9) | 2 Month USG |
| Tufek et al. (2016) [40] | NA | NA | NA | 0 | 0 | 71 (37)* | 186 (56)* | 5 (3)* | 5 (3)* | NA |
| Abreu et al. (2014) [64] | 8 (0-16)^#^ | NA | NA | 1 | 2 | 75 (20-150)^#^ | 210 (80 – 360)^#^ | 8 (6 – 16)^#^ | 2 (1-3)^#^ | 18 (7 – 27)^#^ |
| Moreno Sierra et al. (2010) [65] | NA | NA | Significant Improvement | 0 | 0 | <100 | 80 | 7 | 7 | NA |
| Kural et al. (2009) [2] | NA | NA | NA | NA | NA | NA | 230 | 7 | 7 | NA |
| Magera et al. (2008) [66] | NA | NA | 29 | 0 | 0 | 200 | 300 | 14 | 2 | 14 Days Cystogram |
| Mmeje et al. (2008) [36] | Content | Content | NA | 0 | 0 | 150 | 207 | 9 | 1 | 1 |
| Myer et al. (2007) [38] | NA | NA | 22 (0-300)^#^ | NA | NA | NA | 178  (163 – 235)^#^ | 25 (23 – 32)^#^ | NA | 12 |
| Overall [N=15] | Clustering  5-8 | NA | Clustering  30-60 | 3/120 (2.5%) | 11/120 (9.2%) | Clustering  50-150 | Clustering  120-240 | Clustering  5-15 | Clustering 3-7 | NA |
| *= Mean (SD/Range) | | | | | | # = Median (IQR/Range) | | | | |

(RD: Robotic Diverticulectomy; CT: Computed Tomography; US: Ultrasonography; IPSS: International Prostate Symptom Score; PVR: Postvoid Residual Volume; CD: Clavien Dindo)
