# Supplementary material for: Surgical management of acquired bladder diverticula in adult men: a scoping review
Source: World J Urol. 2026 Jul 31;44(1):537. doi: 10.1007/s00345-026-06633-5 (PMC13427780; doi:10.1007/s00345-026-06633-5)
Supplement: Supplementary file 4 — Supplementary Material 4 [file 345_2026_6633_MOESM3_ESM.docx]

**Supplementary Table 5. Sensitivity analysis comparing the full cohort with the eligibility-refined cohort by surgical approach**

| **Parameter** | **Transurethral** | **Laparoscopic** | **Robotic** | **Open** |
| --- | --- | --- | --- | --- |
| **Patients (n)** | 112 / 112 | 93 / 92 | 120 / 115 | 133 / 132 |
| **Operative time (min)** | 86 / 86 | 196 / 196 | 165 / 165 | 148 / 148 |
| **Catheter (days)** | 9.8 / 9.8 | 9.1 / 9.1 | 7.1 / 7.1 | 14.9 / 14.9 |
| **Length of stay (days)** | 5.6 / 5.6 | 6.2 / 6.2 | 3.6 / 3.6 | 10.8/ 10.8 |
| **Qmax change (mL/s)** | +8.4 / +8.4 | +11.3 / +11.3 | +12.2 / +12.2 | +15.2 / +15.2 |

#- Each cell presents the full-cohort value / refined-cohort value.

The refined cohort excludes 7 non-eligible patients (3 oncologic-primary, 3 female, 1 paediatric). Excluding the seven non-eligible patients did not meaningfully change any pooled estimate. Operative time differed by ≤0.3 minutes, catheter duration and length of stay differed by ≤0.1 days across approaches. Estimated blood loss, functional improvement, complication and clinical-success rates were similarly unaffected.
