# Supplementary material for: Surgical management of acquired bladder diverticula in adult men: a scoping review
Source: World J Urol. 2026 Jul 31;44(1):537. doi: 10.1007/s00345-026-06633-5 (PMC13427780; doi:10.1007/s00345-026-06633-5)
Supplement: Supplementary file 5 — Supplementary Material 5 [file 345_2026_6633_MOESM4_ESM.docx]

**Supplementary Table 2a. Baseline Characteristics and Demographics of Transurethral Group**

| **Author (Year)** | **Type of Study** | **Sample Size (BD)** | **Sample Size (BPO)** | | **Treatment Approach (BPO)** | **Diagnostic** | **Age (years)** | **Prostate Size (cc)** | **Indication of Surgery** | **BD Size (cm)** | | **IPSS** | **Qmax (ml/sec)** | **PVR (ml)** |
| --- | --- | --- | --- | --- | --- | --- | --- | --- | --- | --- | --- | --- | --- | --- |
| Rathinam et al. (2025) [10] | CS | 6 | 6^$^ | | HoLEP | US/CT | 72 (7.46)* | 91 (43)* | RUTI, SBD, Ipsilateral HUN | 8.57 (5.28)* | | 22.8 (5.9)* | 3.1 (1.75)* | 372.8 (288.3)* |
| Pacella et al. (2019) [27] | CS | 39 | 39^$^ | | TUIP  TURP | Cystogram / US+CT | 69.4 (9.8)* | NA | NA | NA | | NA | NA | NA |
| Pacella et al (2018) [8] [Transurethral Subgroup] | RC | 20 | 20^$^ | | TURP | Cystogram / CT | 65 (59.5 - 70)# | 41.5  (30.2 -47.7)^#^ | Diverticula  > 4cm | 7  (5.3 - 7.9)^#^ | | NA | NA | NA |
| Pham et al. (2016) [9] | CR | 1 | 1^$^ | | TURP | Cystoscopy | 50 | NA | LUTS | NA | | NA | NA | NA |
| Okamura et al. (1999) [51] | CS | 2 | 2^$^ | | TURP | Cystoscopy | 75, 76 | NA | LUTS | NA | | NA | NA | 400, 800 |
| Clayman et al. (1984) [26]  [Transurethral Subgroup] | RC | 11 | 5^$^ | | TURP | Cystogram | 65 (TD Alone)* 66 (TD and TURP)* | NA | LUTS, RUTI | NA | | NA | NA | 62 (TD) 185 (TD+ TURP)* |
| Vitale et al. (1979) [28] | CS | 6 | 1^$^  2^^^ | | TURP | Cystogram | Range: 65 - 74 | NA | RUTI, Poor Emptying | NA | | NA | NA | NA |
| Posta (1977) [25] | CS | 10 | 8^$^  2^^^ | | TURP | Cystogram | 71.7 (Range: 64 - 79)* | NA | LUTS | NA | | NA | NA | NA |
| Orandi (1977) [24] | CS | 17 | 17^$^ | | TURP | Cystogram | NA | NA | NA | NA | | NA | NA | NA |
| Overall [N=9]  (1977-1999)+(2016-2025) | 2 RC  6 CS  1 CR | 112 | 103  99^$^ | | 89 TURP  8 TUIP  6 HoLEP | 6 Cystogram  2 Cystoscopy  3 CT / 2 US | Clustering 65-75 | NA | 4 LUTS  3 RUTI | Clustering  7-8.5 | | NA | NA | Mostly > 150 |
| * = Mean (SD/Range) | | | | # = Median (IQR/Range) | | | | $ = Concomitant | | | ^ = Prior/Staged | | | |

(RC: Retrospective comparative; CS: Case series; CR: Case report; BD: Bladder diverticulum; BPO: Benign prostatic obstruction; TURP: Transurethral Resection of the Prostate; HoLEP: Holmium Laser Enucleation of the Prostate; TUIP: Transurethral Incision of the Prostate; CT: Computed Tomography; US: Ultrasonography; RUTI: Recurrent Urinary Tract Infection; SBD; Bladder Stone; LUTS: Lower Urinary Tract Symptoms; HUN: Hydroureteronephrosis; IPSS: International Prostate Symptom Score; PVR: Postvoid Residual Volume)

**Supplementary Table 2b. Perioperative and Postoperative Outcomes of Transurethral Group**

| **Author (Year)** | **BD Size** | **IPSS** | **Qmax (ml/sec)** | **PVR (ml)** | **Major Complications (CD≥3)** | **Minor Complications (CD≤2)** | **Blood Loss** | **Operative Time (mins)** | **Duration of Catheter (days)** | **Length of Stay (days)** | **Follow Up** |
| --- | --- | --- | --- | --- | --- | --- | --- | --- | --- | --- | --- |
| Rathinam et al. (2025) [10] | NA | NA | NA | NA | 0 | 1 | 1.76 (0.82)  Hgb Drop* | 217 (43.45)* | NA | 18 (0.81) Hours* | CT Cystography at 6-8 weeks |
| Pacella et al. (2019) [27] | 30 (76.9%) Success | 62%  (46 - 74%) improvement^#^ | NA | NA | 0 | 2 | NA | 65 (21.9)* | 10  (8 - 12)^#^ | 5 (4 - 6)^#^ | 3 Months Cystogram, USG |
| Pacella et al (2018) [8] [Transurethral Subgroup] | 15/20 (75%) Success | 53%  (50 - 68.2%) Improvement^#^ | NA | NA | 0 | 0 | 0.8 (IQR: 0.72 - 0.9) Hgb Drop^#^ | 62.5  (43.5 - 87.5)* | 12.5  (4 - 15)^#^ | 5 (4 - 6)^#^ | 3 Months Cystogram, USG |
| Pham et al. (2016) [9] | Near Complete Resolution | NA | 11.5 | NA | 0 | 0 | NA | NA | NA | NA | 6 Months |
| Okamura et al. (1999) [51] | 2.8, 3.9 | NA | NA | NA | 0 | 0 | NA | 242, 311 | NA | NA | 19, 20 Months USG, Cystoscopy |
| Clayman et al. (1984) [26]  [Transurethral Subgroup] | Resolution: 5/6 (83.3%) (TD)  2/5 (40%) (TD+TURP) | NA | NA | 8 (TD), 32 (TD+TURP)* | 0 | 0 (TD),  1 (TD+TURP) | <50ml (TD)  460ml (TD+TURP)* | 75 (TD)  114(TD+TURP) | Range:  3-5 | 8 (TD)  9(TD+TURP) | 2 - 19 Months |
| Vitale et al. (1979) [28] | Resolution: 3/6 (50%) | NA | NA | NA | 0 | 0 | NA | NA | Range:  3-5 | NA | NA |
| Posta (1977) [25] | Resolution: 8/10 (80%) | NA | NA | NA | 0 | 0 | 4/10 (40%) intensive 3/10 (30%) Medium | NA | NA | NA | NA |
| Orandi (1977) [24] | Resolution: 5/17 (29%) | NA | NA | NA | 0 | 0 | NA | NA | NA | NA | 1-12 Months Cystogram |
| Overall [N=9] | Clustering 70-80% Success | Clustering  50-65% improvement | NA | NA | 0 | 4/112 (3.6%) | Clustering 1-1.25 Hgb Drop ~ 500ml | Clustering  60-75 and 200 -300 intervals | Clustering 3-5 and  10-12.5 intervals | Clustering  6-8  Interval | Clustering USG Cystogram/graphy |
| * = Mean (SD/Range) | | | | | # = Median (IQR/Range) | | | | | | |

(IPSS: International Prostate Symptom Score; PVR: Postvoid Residual Volume; CD: Clavien Dindo)
