# Supplementary material for: Surgical management of acquired bladder diverticula in adult men: a scoping review
Source: World J Urol. 2026 Jul 31;44(1):537. doi: 10.1007/s00345-026-06633-5 (PMC13427780; doi:10.1007/s00345-026-06633-5)
Supplement: Supplementary file 6 — Supplementary Material 6 [file 345_2026_6633_MOESM5_ESM.docx]

**Supplementary Table 3a. Baseline Characteristics and Demographics of Laparoscopic Group**

| **Author (Year)** | **Type of Study** | **Sample Size (BD)** | **Sample Size (BPO)** | | **Treatment Approach (BPO)** | **Diagnostic** | **Age** | **Baseline PSA** | **Prostate Size** | **Indication of Surgery** | **BD Size** | | **IPSS** | **Qmax** | **PVR** |
| --- | --- | --- | --- | --- | --- | --- | --- | --- | --- | --- | --- | --- | --- | --- | --- |
| Kang et al. (2020) [52] | CS | 12 | 12^$^ | | TUEP | Cystoscopy | 77.5 (3.5)* | 4.9 (3.4) | 137.3 (96.3) | NA | NA | | NA | 5.1 (1.4)* | NA |
| Iscaife et al. (2018) [11]  [ALL Patients Including OD] | RC | 18  [LD] | 18^$^ | | NA | CT | 67.2 (12.7) AUR(+) 68.5 (10.4) AUR (-)* | 9.6 (21) AUR (+) 3.5 (5.5) AUR (-)^#^ | 70.7 (58.2) AUR(+) 50.1 (30.2) AUR(-)* | LUTS | 6.8 (2.5) AUR (+) 4.5 (2) AUR(-)* | | NA | 7.9 (7.1)  AUR (+)  6.1 (4.9)  AUR (-)* | NA |
| Pacella et al. (2018)  [LD Subgroup] [8] | RC | 13 | 13^$^ | | TURP | Cystogram/CT | 63 (51.5-70.5)^#^ | NA | 45  (25 – 55)# | Diverticula > 4cm | 7  (6.3-8.4)^#^ | | NA | NA | NA |
| Yu et al. (2016) [53] | CR | 1 | 1^$^ | | TUEP | CT + US | 82 | 3.9 | 60 | LUTS | 12 | | NA | 4.3 | 370 |
| Magdy et al. (2016) [54] | CS | 4 | 0 | | NA | US | 71.7 (63 - 86)* | NA | NA | RUTI  High PVR | 4.4  (2.3 - 7)* | | NA | 17.27  (15.8-19.2)* | 147.5  (120 -180)* |
| Hora et al. (2015) [3] | CS | 14 | 8^$^  5^^^ | | 8x PVP  1x LP  4x TURP | CT Cystography | 66.5 (5.5)* | 2.9 (1.8)* | 44.5 (14.7)* | NA | NA | | NA | NA | NA |
| Roslan et al. (2012) [55] | CS | 3 | 3 | | Medical | IVP / US | 67 (62 - 76)* | NA | 34.3  (28 - 40)* | LUTS RUTI | 5.3  (5-6)* | | NA | 17.7  (15 - 21)* | 110  (80 - 150)* |
| Shah et al. (2006) [32] | CS | 3 | 3^$^ | | HoLEP | RUG / US | 66  (56 - 78)* | NA | 56.4  (22 - 80)* | LUTS HUN | NA | | 13  (11-16)* | 4.9  (2.4 -6.5)* | 997  (576 - 1244)* |
| Porpiglia et al. (2004)  [LD Subgroup] [31] | RC | 12 | 12^$^ | | TURP | US / CT /  RUG / VCUG | NA | NA | 30  (15 - 40)* | LUTS RUTI | NA | | NA | NA | NA |
| Faramarzi-Roques et al. (2004) [56] | CS | 5 | 4^$^  1^^^ | | TURP | Cystoscopy | 64.2  (55 - 76)* | NA | NA | LUTS | NA | | NA | NA | NA |
| Khonsari et al. (2004) [57] | CR | 1 | NA | | NA | IVP + Cystogram | 49 | NA | NA | NA | >1000cc | | NA | 7.7 | NA |
| Juan et al. (2004) [58] | CR | 1 | 1^^^ | | TURP | Cystogram | 85 | NA | NA | NA | NA | | NA | NA | NA |
| Iselin et al. (1996)  [LD Subgroup] [48] | CS | 2 | 2^$^ | | TURP | Cystoscopy / Cystography | NA | NA | NA | NA | 4.5  (4 - 5)* | | NA | NA | NA |
| Nadler et al. (1995) [59] | CR | 1 | 1^$^ | | TUIP | Cystoscopy | 56 | 4 | 20 | Elective | 300cc | | NA | NA | NA |
| Jarret et al. (1995) [30] | CR | 1 | 1^^^ | | TURP | Video UDS | 84 | NA | NA | RUTI | NA | | NA | NA | NA |
| Das (1992) [60] | CR | 1 | 1^^^ | | TURP | Urogram /  X-Ray | 72 | NA | NA | RUTI  LUTS | 11 | | NA | NA | NA |
| Parra et al. (1992) [29] | CR | 1 | 1^^^ | | TURP | Cystoscopy | 87 | NA | NA | RUTI LUTS | NA | | NA | NA | NA |
| Overall [N= 17]  (1992 – 2006)+(2015-2018) | 3 RC  7 CS  7 CR | 93 | 86  74^$^ | | 46 TURP  13 TUEP | 5 Cystoscopy  5 CT / 5 US  3 Cystogram | Clustering  65-75 | Clusters  3-4.5 | Clustering  30-70 | 8 LUTS  6 RUTI | Clusters  4.5-7 | | NA | Clustering  5-10 | Mostly >100 |
| *=Mean (SD/Range) | | | | #=Median (IQR/Range) | | | | $ = Concomitant | | | | ^ = Prior/Staged | | | |

(LD: Laparoscopic Diverticulectomy; RC: Retrospective comparative; CS: Case series; CR: Case report; BPO: Benign prostatic obstruction; TURP: Transurethral Resection of the Prostate; US: Ultrasonography; RUG: Retrograde Urethrography; IVP: Intravenous Pyelography; RUTI: Recurrent Urinary Tract Infection; SBD; Bladder Stone; LUTS: Lower Urinary Tract Symptoms; HUN: Hydroureteronephrosis; AUR; Acute Urinary Retention; IPSS: International Prostate Symptom Score; PVR: Postvoid Residual Volume)

**Supplementary Table 3b. Perioperative and Postoperative Outcomes of Laparoscopic Group**

| **Author (Year)** | **BD Size** | **IPSS** | **Qmax** | **PVR** | **Major Complications (CD≥3)** | **Minor Complications (CD≤2)** | **Blood Loss** | **Operative Time (mins)** | **Duration of Catheter (days)** | **Length of Stay (days)** | **Follow Up** |
| --- | --- | --- | --- | --- | --- | --- | --- | --- | --- | --- | --- |
| Kang et al. (2020) [52] | NA | NA | 12.8 (2.3)* | NA | 0 | 1 | 52.1 (14.9)* | 214.2 (69)* | 14 | 9 (3.5) | 3.5 (3.0 - 5.3)# |
| Iscaife et al. (2018) [11]  [ALL Patients Including OD] | NA | NA | 24.8 (13.8) AUR (+) 19.6 (9) AUR (-)* | NA | NA | NA | NA | NA | NA | NA | NA |
| Pacella et al. (2018)  [LD Subgroup] [8] | 13/13 (100%) Success | 60% (56-69.6) Improvement# | NA | NA | 2 | 0 | 0.9 (0.75 - 0.95) Hgb Drop* | 185  (130 - 225)^#^ | 7 (5 - 13.5)# | 4  (3 - 6)# | 3 Months via CT Cystography, US |
| Yu et al. (2016) [53] | NA | NA | NA | NA | 0 | 0 | NA | 126 | 10 | NA | NA |
| Magdy et al. (2016) [54] | NA | NA | NA | NA | 1 | 0 | <50ml | 134.25 (44.92)* | 10 | 10 | Cystogram POD 10, 11.6 (10.4 - 12)* |
| Hora et al. (2015) [3] | NA | NA | NA | NA | 0 | 0 | NA | 165 (48.5)* | 6 | NA | `NA |
| Roslan et al. (2012) [55] | NA | NA | NA | NA | 0 | 0 | Minimal | 128  (80 - 175)* | 5.6 (4 - 7)* | 3 (2- 4)* | 3 months USG, Cystoscopy |
| Shah et al. (2006) [32] | NA | 6 (3 - 8)* | 10.4  (7 - 14.5)* | 164  (28 - 286)* | 0 | 0 | 1.13 (0.8 - 1.4) Hgb Drop* | 63.3  (40 - 90) HoLEP 246.6  (210 - 300) LD* | 14 | 2.8 hours (48 - 80)* | Cystogram POD 14, 6 months AUASI, Uroflowmetry, PVR |
| Porpiglia et al. (2004)  [LD Subgroup] [31] | NA | NA | 18.4  (16 - 22)* | NA | NA | NA | 2.5 (2 - 4)  Hgb Drop* | 239  (180 -300)* | 7 | NA | NA |
| Faramarzi-Roques et al. (2004) [56] | NA | NA | NA | NA | 0 | 0 | 150 (80 - 200)* | 160  (120 -130) | 5 (3 - 7)* | 5 (4 - 6) | 2 Months, US |
| Khonsari et al. (2004) [57] | NA | NA | NA | NA | 0 | 0 | NA | NA | 5 | NA | 3 Months |
| Juan et al. (2004) [58] | NA | NA | NA | NA | NA | NA | NA | NA | 8 | 8 | NA |
| Iselin et al. (1996)  [LD Subgroup] [48] | NA | NA | NA | NA | 0 | 0 | 6.5% (2- 15) Hct Drop* | 270  (240-300)* | 10 | 4.5  (4 - 5)* | 1 Month |
| Nadler et al. (1995) [59] | Complete Resolution | NA | NA | NA | 0 | 0 | 100cc | 460 | 15 | 2 | 2 months cystoscopy, US |
| Jarret et al. (1995) [30] | NA | NA | NA | NA | 0 | 0 | NA | 390 | 9 | 3 | 10 |
| Das (1992) [60] | NA | NA | NA | NA | NA | NA | NA | 165 | NA | NA | NA |
| Parra et al. (1992) [29] | NA | NA | NA | NA | 0 | 0 | NA | 290 | 8 | 8 | 6 weeks |
| Overall [N= 17] | Mostly Resolved | NA | Clustering 12.5 – 20 | NA | 3/93 (3.2%) | 1/93 (1.1%) | Heterogeneous | Clustering  120 - 240 | Clustering 5-10 | Clustering  3-5 | USG + Cystogram  Cystography  3-6 Months |
| *=Mean (SD/Range) | | | | | | #=Median (IQR/Range) | | | | | |

(LD: Laparoscopic Diverticulectomy; CT: Computed Tomography; US: Ultrasonography; Acute Urinary Retention; IPSS: International Prostate Symptom Score; PVR: Postvoid

Residual Volume; CD: Clavien Dindo)
