# Supplementary material for: Surgical management of acquired bladder diverticula in adult men: a scoping review
Source: World J Urol. 2026 Jul 31;44(1):537. doi: 10.1007/s00345-026-06633-5 (PMC13427780; doi:10.1007/s00345-026-06633-5)
Supplement: Supplementary file 7 — Supplementary Material 7 [file 345_2026_6633_MOESM6_ESM.docx]

**Supplementary Table 6. IHE Quality Appraisal Checklist**

| **Study** | **Design** | **N** | **Approach** | **1** | **2** | **3** | **4** | **5** | **6** | **7** | **8** | **9** | **10** | **11** | **12** | **13** | **14** | **15** | **16** | **17** | **18** | **19** | **20** |
| --- | --- | --- | --- | --- | --- | --- | --- | --- | --- | --- | --- | --- | --- | --- | --- | --- | --- | --- | --- | --- | --- | --- | --- |
| Liu et al. (2021) | RC | 26 | Robotic/Open | Y | N | P | Y | Y | Y | P | Y | Y | Y | NA | Y | Y | Y | Y | Y | Y | Y | Y | Y |
| Iscaife et al. (2018) | RC | 47 | Open/Laparoscopic | Y | N | N | P | Y | P | P | Y | Y | Y | NA | Y | Y | Y | P | P | Y | Y | Y | P |
| Michelotti et al. (2008) | CR | 1 | Open | Y | NA | NA | NA | Y | NA | NA | Y | Y | P | NA | Y | Y | NA | P | NA | NA | Y | Y | P |
| Porpiglia et al. (2004) | RC | 25 | Laparoscopic/Open | Y | N | N | P | Y | P | P | Y | Y | Y | NA | Y | Y | Y | P | P | Y | Y | Y | P |
| Iselin et al. (1996) | CS | 6 | Laparoscopic/Open | Y | N | N | U | P | P | P | Y | P | P | NA | P | P | N | P | U | P | P | Y | N |
| Gong and Issa (1996) | CR | 1 | Open | Y | NA | NA | NA | P | NA | NA | Y | P | P | NA | Y | P | NA | P | NA | NA | P | Y | N |
| Jarow and Brendler (1988) | CR | 1 | Open | Y | NA | NA | NA | P | NA | NA | Y | P | P | NA | Y | P | NA | P | NA | NA | P | Y | N |
| Clayman et al. (1984) | RC | 41 | Transurethral/Open | Y | N | N | P | Y | P | P | Y | Y | Y | NA | Y | Y | Y | P | P | Y | Y | Y | P |
| Firstater and Farkas (1977) | CS | 48 | Open | Y | N | N | U | P | P | P | Y | P | P | NA | P | P | N | P | U | P | P | Y | N |
| Rathinam et al. (2025) | CS | 6 | Transurethral | Y | P | N | Y | Y | Y | P | Y | Y | Y | NA | Y | Y | Y | Y | Y | Y | Y | Y | Y |
| Pacella et al. (2019) | CS | 39 | Transurethral | Y | P | N | Y | Y | Y | P | Y | Y | Y | NA | Y | Y | Y | Y | Y | Y | Y | Y | Y |
| Pacella et al. (2018) | RC | 33 | Transurethral/Laparoscopic | Y | N | P | Y | Y | Y | P | Y | Y | Y | NA | Y | Y | Y | Y | Y | Y | Y | Y | Y |
| Pham et al. (2016) | CR | 1 | Transurethral | Y | NA | NA | NA | Y | NA | NA | Y | Y | P | NA | Y | Y | NA | P | NA | NA | Y | Y | P |
| Okamura et al. (1999) | CS | 2 | Transurethral | Y | N | N | U | P | P | P | Y | P | P | NA | P | P | N | P | U | P | P | Y | N |
| Vitale and Woodside (1979) | CS | 6 | Transurethral | Y | N | N | U | P | P | P | Y | P | P | NA | P | P | N | P | U | P | P | Y | N |
| Posta (1977) | CS | 10 | Transurethral | Y | N | N | U | P | P | P | Y | P | P | NA | P | P | N | P | U | P | P | Y | N |
| Orandi (1977) | CS | 17 | Transurethral | Y | N | N | U | P | P | P | Y | P | P | NA | P | P | N | P | U | P | P | Y | N |
| Kang et al. (2020) | CS | 12 | Laparoscopic | Y | P | N | Y | Y | Y | P | Y | Y | Y | NA | Y | Y | Y | Y | Y | Y | Y | Y | Y |
| Yu et al. (2016) | CR | 1 | Laparoscopic | Y | NA | NA | NA | Y | NA | NA | Y | Y | P | NA | Y | Y | NA | P | NA | NA | Y | Y | P |
| Magdy et al. (2016) | CS | 4 | Laparoscopic | Y | N | N | P | Y | P | P | Y | Y | P | NA | Y | Y | P | Y | P | P | Y | Y | P |
| Hora et al. (2015) | CS | 14 | Laparoscopic | Y | N | N | P | Y | P | P | Y | Y | P | NA | Y | Y | P | Y | P | P | Y | Y | P |
| Roslan et al. (2012) | CS | 3 | Laparoscopic | Y | N | N | P | Y | P | P | Y | Y | P | NA | Y | Y | P | Y | P | P | Y | Y | P |
| Shah et al. (2006) | CS | 3 | Laparoscopic | Y | N | N | P | Y | P | P | Y | Y | P | NA | Y | Y | P | Y | P | P | Y | Y | P |
| Faramarzi-Roques et al. (2004) | CS | 5 | Laparoscopic | Y | N | N | P | Y | P | P | Y | Y | P | NA | Y | Y | P | Y | P | P | Y | Y | P |
| Khonsari et al. (2004) | CR | 1 | Laparoscopic | Y | NA | NA | NA | Y | NA | NA | Y | Y | P | NA | Y | Y | NA | P | NA | NA | Y | Y | P |
| Juan et al. (2004) | CR | 1 | Laparoscopic | Y | NA | NA | NA | Y | NA | NA | Y | Y | P | NA | Y | Y | NA | P | NA | NA | Y | Y | P |
| Nadler et al. (1995) | CR | 1 | Laparoscopic | Y | NA | NA | NA | P | NA | NA | Y | P | P | NA | Y | P | NA | P | NA | NA | P | Y | N |
| Jarrett et al. (1995) | CR | 1 | Laparoscopic | Y | NA | NA | NA | P | NA | NA | Y | P | P | NA | Y | P | NA | P | NA | NA | P | Y | N |
| Das (1992) | CR | 1 | Laparoscopic | Y | NA | NA | NA | P | NA | NA | Y | P | P | NA | Y | P | NA | P | NA | NA | P | Y | N |
| Parra et al. (1992) | CR | 1 | Laparoscopic | Y | NA | NA | NA | P | NA | NA | Y | P | P | NA | Y | P | NA | P | NA | NA | P | Y | N |
| Gibson et al. (2024) | CS | 18 | Robotic | Y | P | N | Y | Y | Y | P | Y | Y | Y | NA | Y | Y | Y | Y | Y | Y | Y | Y | Y |
| Orsini et al. (2024) | CS | 4 | Robotic | Y | P | N | Y | Y | Y | P | Y | Y | Y | NA | Y | Y | Y | Y | Y | Y | Y | Y | Y |
| Giannarini et al. (2022) | CS | 16 | Robotic | Y | P | N | Y | Y | Y | P | Y | Y | Y | NA | Y | Y | Y | Y | Y | Y | Y | Y | Y |
| Develtere et al. (2022) | CS | 23 | Robotic | Y | P | N | Y | Y | Y | P | Y | Y | Y | NA | Y | Y | Y | Y | Y | Y | Y | Y | Y |
| Agarwal and Krambeck (2018) | RC | 2 | Robotic/Open | Y | N | N | P | Y | P | P | Y | Y | Y | NA | Y | Y | Y | P | P | Y | Y | Y | P |
| Ashton et al. (2018) | CS | 3 | Robotic | Y | N | N | P | Y | P | P | Y | Y | P | NA | Y | Y | P | Y | P | P | Y | Y | P |
| Cacciamani et al. (2018) | CS | 6 | Robotic | Y | P | N | Y | Y | Y | P | Y | Y | Y | NA | Y | Y | Y | Y | Y | Y | Y | Y | Y |
| Tufek et al. (2016) | CS | 9 | Robotic | Y | N | N | P | Y | P | P | Y | Y | P | NA | Y | Y | P | Y | P | P | Y | Y | P |
| de Castro Abreu et al. (2014) | CS | 10 | Robotic | Y | N | N | P | Y | P | P | Y | Y | P | NA | Y | Y | P | Y | P | P | Y | Y | P |
| Moreno Sierra et al. (2010) | CR | 1 | Robotic | Y | NA | NA | NA | Y | NA | NA | Y | Y | P | NA | Y | Y | NA | P | NA | NA | Y | Y | P |
| Kural et al. (2009) | CR | 1 | Robotic | Y | NA | NA | NA | Y | NA | NA | Y | Y | P | NA | Y | Y | NA | P | NA | NA | Y | Y | P |
| Magera et al. (2008) | CR | 1 | Robotic | Y | NA | NA | NA | Y | NA | NA | Y | Y | P | NA | Y | Y | NA | P | NA | NA | Y | Y | P |
| Mmeje et al. (2008) | CR | 1 | Robotic | Y | NA | NA | NA | Y | NA | NA | Y | Y | P | NA | Y | Y | NA | P | NA | NA | Y | Y | P |
| Myer and Wagner (2007) | CS | 5 | Robotic | Y | N | N | P | Y | P | P | Y | Y | P | NA | Y | Y | P | Y | P | P | Y | Y | P |

Y=yes; P=partially met; N=no; U=unclear; NA=not applicable. Item 11 (blinded outcome assessment) was considered not applicable for this evidence base. Items: 1 objective stated; 2 prospective design; 3 multicentre; 4 consecutive recruitment; 5 patient characteristics described; 6 eligibility criteria stated; 7 similar point in disease; 8 intervention described; 9 co-interventions described; 10 outcomes established a priori; 11 blinded outcome assessment; 12 appropriate outcome measures; 13 outcomes measured before/after; 14 appropriate statistical analysis; 15 follow-up sufficiently long; 16 losses to follow-up reported; 17 estimates of variability reported; 18 adverse events reported; 19 conclusions supported; 20 competing interests/funding reported.
