# Supplementary material for: Surgical management of acquired bladder diverticula in adult men: a scoping review
Source: World J Urol. 2026 Jul 31;44(1):537. doi: 10.1007/s00345-026-06633-5 (PMC13427780; doi:10.1007/s00345-026-06633-5)
Supplement: Supplementary file 8 — Supplementary Material 8 [file 345_2026_6633_MOESM7_ESM.docx]

**Supplementary Figure 1. PRISMA Diagram**

**Identification**

Studies included
(n =44)

Full-text articles assessed for eligibility
(n =99)

Articles sought for retrieval based on tittle and abstract

(n = 99)

Records identified through PUBMED(MEDLINE) database
(n = 1147)

Records excluded
(n = 1048)

**Screening**

Full-text articles excluded (n= 55)

**Reasons**

- No surgical treatment of BD
- Included ≥ 10 % female
- Included ≥ 10 % pediatric
- Included ≥10 % oncological

**Eligibility**

**Included**

Transurethral = 7

Laparoscopic = 13

Robotic = 13

Open = 5

Combined = 6
