# Supplementary figures and images for: Surgical management of acquired bladder diverticula in adult men: a scoping review
Source: World J Urol. 2026 Jul 31;44(1):537. doi: 10.1007/s00345-026-06633-5 (PMC13427780; doi:10.1007/s00345-026-06633-5)

**Supplementary Figure 4. Approach to Acquired Benign BD Algorithm**

**
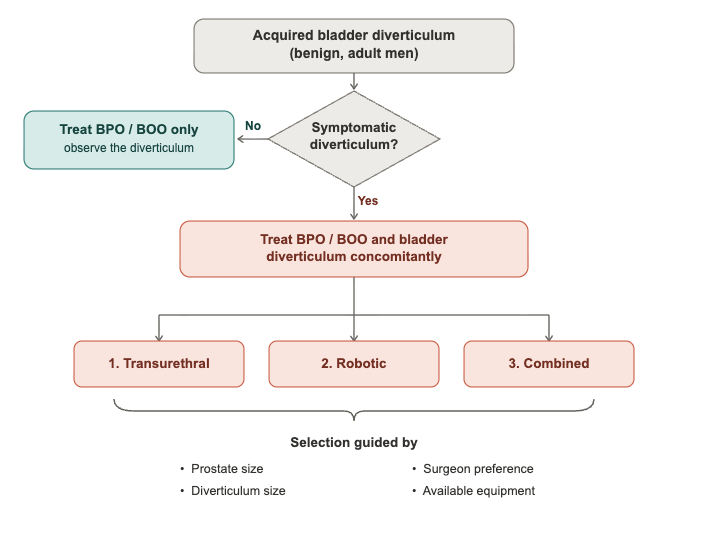
**

Supplement: Supplementary file 9 — Supplementary Material 9 [file 345_2026_6633_MOESM8_ESM.docx]

**Supplementary Figure 2. Cumulative Trends of BD Literature by Surgical Approach**


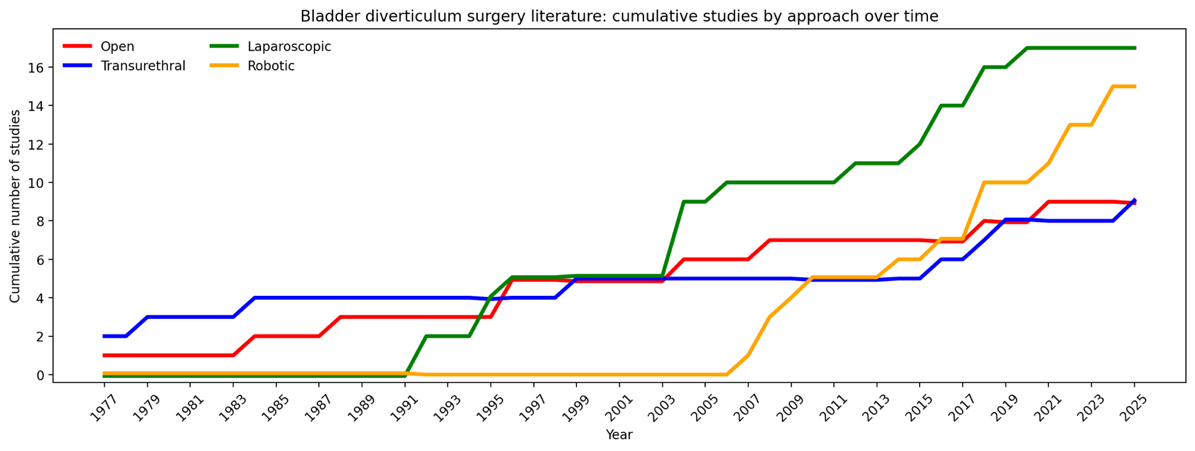

Supplement: Supplementary file 10 — Supplementary Material 10 [file 345_2026_6633_MOESM9_ESM.docx]
